# Durable disease control following multidisciplinary treatment including pembrolizumab in *POLE*-mutated dedifferentiated endometrial carcinoma with brain metastases: A case report

**DOI:** 10.1097/MD.0000000000050714

**Published:** 2026-09-18

**Authors:** Takumi Ito, Jumpei Yoshida, Akari Iwakoshi, Keiji Sugiyama, Rieko Nishimura, Tomoko Inaba, Masashi Sanada, Chiyoe Kitagawa

**Affiliations:** a Department of Medical Oncology, NHO Nagoya Medical Center, Nagoya, Aichi, Japan; b Department of Medical Oncology, Cancer Institute Hospital of Japanese Foundation for Cancer Research, Tokyo, Japan; c Department of Pathology, NHO Nagoya Medical Center, Nagoya, Aichi, Japan; d Department of Gynecology, NHO Nagoya Medical Center, Nagoya, Aichi, Japan; e Clinical Research Center, NHO Nagoya Medical Center, Nagoya, Aichi, Japan.

**Keywords:** case report, Dedifferentiated endometrial carcinoma, Immunotherapy, *POLE* mutation

## Abstract

**Rationale:**

Dedifferentiated endometrial carcinoma (DEC) is a rare and aggressive subtype of endometrial cancer comprising undifferentiated carcinoma and a low-grade endometrioid component. Although *POLE*-mutated tumors usually show favorable outcomes owing to their ultramutated and immunogenic nature, the clinical relevance of *POLE* mutations in DEC remains unclear. In particular, the therapeutic effects of immune checkpoint inhibitors in *POLE*-mutated DEC with brain metastases have not been well characterized.

**Patient concerns:**

Here, we report the case of a 58-year-old woman with *POLE*-mutated DEC who presented with headache, dizziness, and generalized weakness due to multiple brain and pulmonary metastases. The clinical course, pathological and genomic findings, multidisciplinary treatment, and treatment outcomes were evaluated.

**Diagnoses:**

Endometrial biopsy confirmed dedifferentiated carcinoma. Genomic profiling identified a pathogenic *POLE* exonuclease domain mutation (V411L) and a high tumor mutational burden (49.7 mutations/Mb).

**Interventions:**

The patient underwent urgent cerebellar resection, followed by radiotherapy, during which a new intracranial lesion developed.

**Outcomes:**

Systemic therapy with carboplatin, paclitaxel, and pembrolizumab resulted in marked regression of uterine and pulmonary lesions. Her symptoms resolved completely, and the Eastern Cooperative Oncology Group performance status improved from 2 to 0. During maintenance pembrolizumab therapy, further tumor regression was observed, and durable disease control was maintained thereafter. At 14 months, the patient remained progression-free without significant adverse events.

**Lessons:**

This case demonstrated a dramatic and sustained response following multidisciplinary treatment, including surgery, radiotherapy, chemotherapy, and immune checkpoint inhibitor therapy, in *POLE*-mutated DEC with brain metastases. This highlights the importance of molecular profiling in identifying patients who may benefit from immunotherapy, even in aggressive and widely metastatic disease.

## 1. Introduction

In 2022, 420,242 patients were newly diagnosed with endometrial cancer (EC), and 97,704 died worldwide. Over the past 30 years, the incidence of EC has increased by 132%, underscoring its considerable clinical impact.^[1]^ Advances in molecular and pathological research have revealed the heterogeneity and clinical diversity of EC. According to the 2020 World Health Organization classification,^[2]^ the most common histological subtype is endometrioid carcinoma, followed by serous, clear-cell, and mucinous carcinomas. Less common entities include undifferentiated and dedifferentiated carcinomas, carcinosarcomas, neuroendocrine carcinomas, mesonephric carcinomas, mesonephric-like carcinomas, and squamous carcinomas.

The Cancer Genome Atlas (TCGA) proposed a molecular classification of EC into 4 subgroups: *POLE*-ultramutated, microsatellite instability (MSI) hypermutated/mismatch repair (MMR)-deficient, copy-number-low, and copy-number-high (serous-like).^[3]^ *POLE* mutations occur in approximately 7%–10% of patients with EC, predominantly in low-grade endometrioid carcinomas. *POLE* encodes the catalytic subunit of DNA polymerase epsilon, which exhibits proofreading activity. Mutations in the exonuclease domain impair this function, resulting in an ultramutated phenotype. Tumors harboring *POLE* mutations are generally associated with favorable prognoses, exhibit an exceptionally high mutational burden, and demonstrate strong immunogenicity, which may confer sensitivity to immune checkpoint inhibitors (ICIs).

Dedifferentiated endometrial carcinoma (DEC) is an extremely rare histological subtype of EC that accounts for approximately 1% of EC cases.^[4]^ It is defined by the coexistence of an undifferentiated carcinoma and a low-grade endometrioid carcinoma component. DEC frequently exhibits MMR deficiency and alterations in the SWI/SNF chromatin-remodeling complex, including mutations in *ARID1A*, *SMARCA4*, and *SMARCB1*.^[5]^ DEC is associated with an exceptionally aggressive clinical course and poor prognosis, and rare cases of brain metastasis have also been reported, indicating that it can present with highly advanced disease.^[4,6]^ Conventional chemotherapy provides only limited benefits, and even in recent pivotal phase III trials of ICIs for EC, undifferentiated or dedifferentiated histologies accounted for only 0.6%–2.5% of enrolled patients,^[7–9]^ and no standard treatment has been established for this subtype. However, the clinical and biological implications of *POLE* mutations in DEC have not been fully elucidated.

Herein, we report a case of *POLE*-mutated DEC with multiple brain metastases that exhibited a highly aggressive clinical course and achieved durable disease control following multidisciplinary treatment including ICI-based therapy.

## 2. Ethical approval

Ethical review and approval were waived by the Institutional Review Board of the Nagoya Medical Center because this manuscript describes a single-patient case report, which is exempt from ethical review according to institutional policy. Written informed consent was obtained from the patient for publication of this case report and accompanying images. A copy of the written consent form is available for review by the Editor-in-Chief of this journal upon request.

## 3. Case presentation

**Figure 1.**
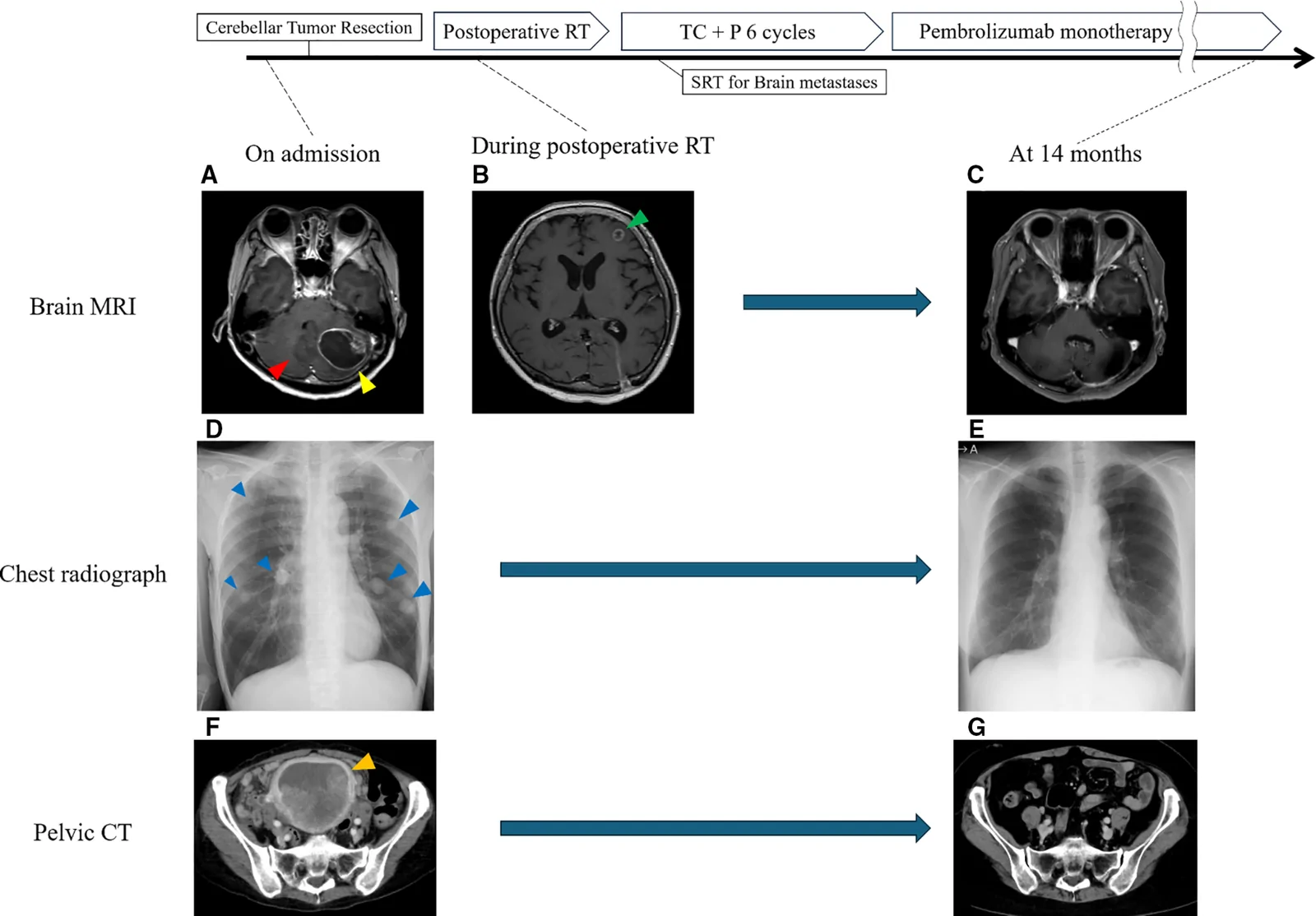
The black horizontal arrow at the top indicates the clinical timeline progressing from left to right, with the boxes showing the timing and duration of treatments and the dashed lines indicating the timing of radiological assessments. (A–C) Brain magnetic resonance imaging (MRI), contrast-enhanced T1-weighted images. (A) On admission, a left cerebellar mass (the yellow arrow) with marked perifocal edema extending toward the brainstem (the red arrow) is observed. (B) During postoperative radiotherapy, 14 days after surgery, a new metastatic lesion appears in the left frontal lobe (the green arrow). (C) At 14 months after treatment initiation, no intracranial recurrence is detected following surgical resection and radiotherapy. (D, E) Chest radiographs. (D) On admission, multiple pulmonary metastases are present (the blue arrows). (E) At 14 months, near-complete interval resolution is observed. (F, G) Contrast-enhanced pelvic computed tomography (CT) images. (F) On admission, an enlarged uterus consistent with the primary tumor is identified (the orange arrow). (G) At 14 months, continued reduction in uterine size is observed. RT = radiotherapy, TC + P = carboplatin, paclitaxel, and pembrolizumab.

A 58-year-old woman presented with a 1-week history of headaches, dizziness, and generalized weakness. Her Eastern Cooperative Oncology Group performance status (ECOG PS) had declined to 2. Contrast-enhanced brain magnetic resonance imaging (MRI) revealed a 3-cm tumor in the left cerebellum (Fig. 1A) and additional lesions in the right temporal lobe. Contrast-enhanced computed tomography (CT) of the abdomen and pelvis revealed an enlarged uterus (Fig. 1F), and chest radiography on admission revealed multiple pulmonary metastases (Fig. 1D).

**Figure 2.**
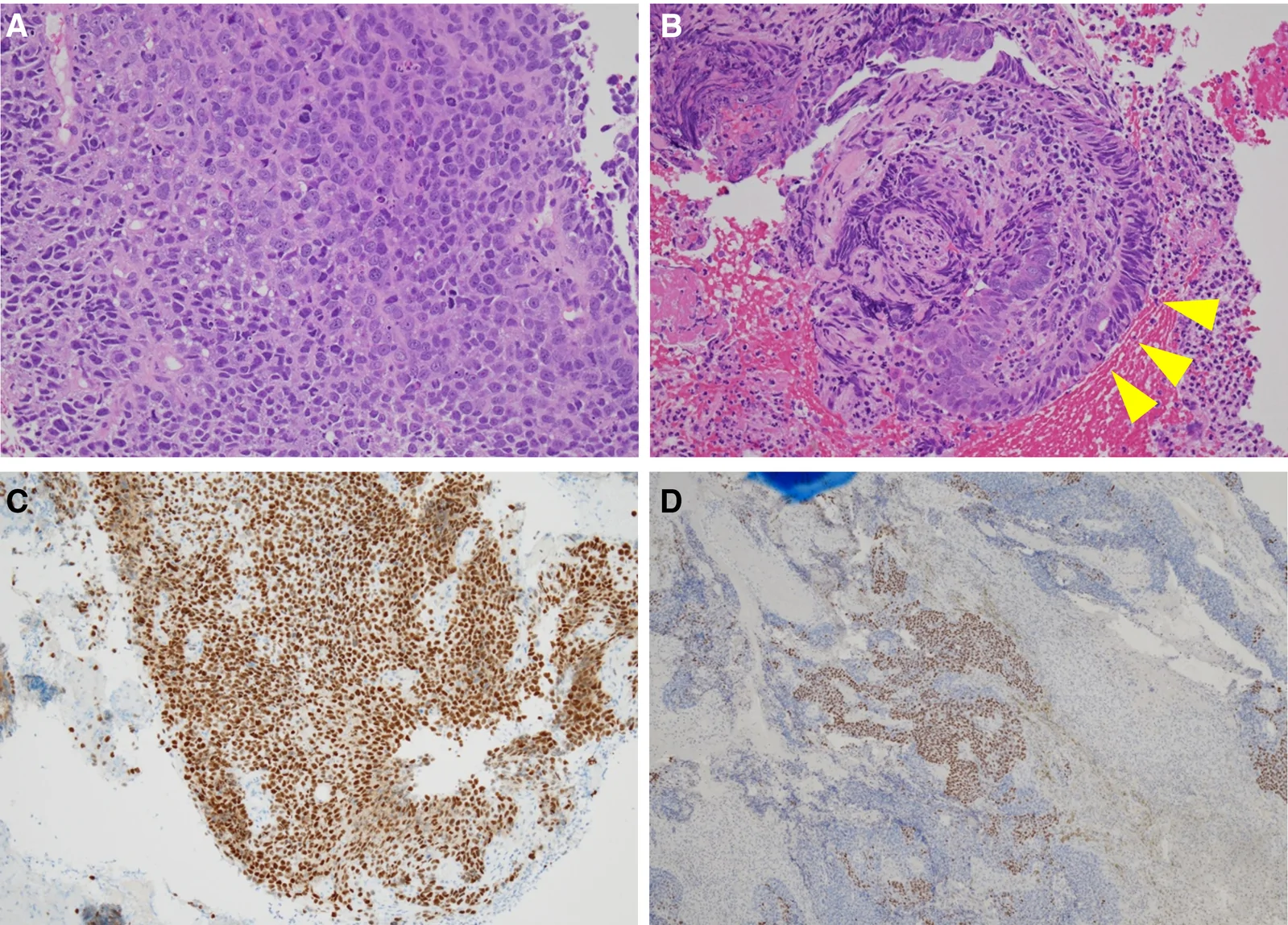
Pathological findings of endometrial biopsy specimens (A–C) and cerebellar metastatic tumor (D). (A) Undifferentiated carcinoma component (H&E, 10×) showing solid nests of atypical epithelial cells with prominent nucleoli and numerous mitotic figures. (B) Differentiated adenocarcinoma component (H&E, 10×), in which definitive histological typing was challenging but atypical glandular structures are observed. (C) Diffuse nuclear positivity for p53 on immunohistochemistry (10×). (D) Cerebellar specimen (H&E, 4×) showing focal positive staining for p40, consistent with squamous differentiation.

Pathological examination of the endometrial biopsy specimen confirmed the diagnosis of DEC (Fig. 2). Comprehensive genomic profiling (CGP) identified a *POLE* V411L mutation (Table 1). The cerebellar metastatic tumor was urgently resected because of the risk of obstructive hydrocephalus and brainstem compression caused by surrounding edema (Fig. 1A). Postoperatively, adjuvant radiotherapy was administered to the resection cavity (21 Gy in seven fractions). During radiotherapy, 14 days after surgery, a new metastatic lesion developed in the left frontal lobe (Fig. 1B), accompanied by the progression of pulmonary metastases. Therefore, systemic therapy was initiated with carboplatin, paclitaxel, and pembrolizumab (paclitaxel 175 mg/m^2^, carboplatin AUC 6, and pembrolizumab 200 mg on day 1 of every 3-week cycle [TC + P]). Concurrent stereotactic radiotherapy was delivered to the residual brain metastases (left frontal lesion: 30 Gy in five fractions; right temporal lesion: 18 Gy in a single fraction). After 3 cycles of TC + P, shrinkage of the primary uterine lesion and pulmonary metastases was observed. Her symptoms completely resolved, and her ECOG PS improved to 0. After completion of six cycles of TC + P, cytotoxic chemotherapy was discontinued, and pembrolizumab monotherapy was continued as maintenance therapy. During pembrolizumab maintenance therapy, several pulmonary metastases showed further regression and eventually achieved a complete response (Fig. 1E), while the primary uterine tumor continued to regress (Fig. 1G). No new intracranial lesions were detected (Fig. 1C), and a partial response was maintained for 14 months, according to the Response Evaluation Criteria in Solid Tumors. Peripheral sensory neuropathy (grade 2) and neutropenia (grade 3) were observed, as assessed using the Common Terminology Criteria for Adverse Events, version 5.0. No other significant adverse events were observed.

**Table 1:** Genomic alterations identified through comprehensive genomic profiling using the GenMineTOP platform.

DNA analysis: somatic mutations			
	Pathogenic variant	protein change	variant allele frequency [%]
1	*ABL1*	R473*	34.2
2	*APC*	R1114*	64.0
3	*ASPM*	*G53E*	36.1
4	*ASPM*	*K2711N*	35.9
5	*ATRX*	E327*	33.9
6	*ATRX*	*R840I*	34.1
7	*BACH1*	*E281K*	38.8
8	*CASP8*	E36*	36.3
9	*FANCM*	*K451N*	38.0
10	*FBXW7*	E255*	46.6
11	*FMN2*	*R1420Q*	29.9
12	*GEN1*	R401*	42.2
13	*GOLGA5*	*R386H*	37.2
14	*GRIN2A*	*S1410L*	40.5
15	*IGF2R*	*D1685N*	43.9
16	*KARS1*	*R205C*	31.1
17	*KAT6A*	*R366Q*	38.1
18	*KDM5C*	*P480L*	59.2
19	*KMT2D (MLL2*)	E3418*	63.0
20	*MAP3K13*	*R401W*	36.8
21	*MAP3K4*	*E262K*	46.4
22	*MELK*	E477*	36.7
23	*MRE11 (MRE11A*)	*E494K*	36.6
24	*MYB*	R294*	12.2
25	*NF1*	*G57S*	34.9
26	*NFE2L2 (NRF2*)	*F37S*	35.4
27	*NOTCH2*	*P1471S*	6.9
28	*PAK3*	*K265N*	37.2
29	*PALLD*	*A509T*	44.2
30	*PIK3CA*	*R88Q*	37.7
31	*PIK3CA*	*R357Q*	38.5
32	*PIK3CG*	*R690I*	36.2
33	*PIK3R1*	R348*	65.9
34	*PINK1*	*R407W*	22.6
35	*POLE*	*V411L*	63.9
36	*PTEN*	*M134I*	60.5
37	*PTEN*	*R142W*	59.4
38	*PTPRT*	*E894K*	36.5
39	*RB1*	E282*	36.2
40	*RB1*	*E322K*	36.3
41	*RXRA*	*R316H*	35.3
42	*TP53*	*F113V*	41.1
43	*TP53*	R213*	35.4
44	*TSC1*	E813*	40.4
45	*UGT1A1*	*R403H*	35.4
46	*UGT1A1*	*A477T*	37.2

Tumor mutation burden: 49.7 muts/Mb.

“*” denotes a stop codon (termination codon).

### 3.1. Pathological findings

An endometrial tumor biopsy revealed solid nests of atypical epithelial cells exhibiting nuclear atypia, prominent eosinophilic nucleoli, numerous mitotic figures, and atypical glandular structures. Although the specimen was extensively necrotic, limiting detailed histological evaluation, the tumor was predominantly undifferentiated, with an admixed adenocarcinoma component (Fig. 2A, B). Immunohistochemically, both the glandular and undifferentiated components were diffusely positive for p53 (Fig. 2C), p16, and vimentin and showed loss of PTEN expression. Estrogen receptors and PAX8 were positive in the glandular component but negative in the undifferentiated component. The expression of all MMR proteins, ARID1A, SMARCB1 (INI1/BAF47), and SMARCA4 (BRG1) was retained. Based on the histomorphology and immunohistochemical profiles, the adenocarcinoma component could not be reliably classified as serous or endometrioid. Overall, the findings were consistent with a diagnosis of DEC. Examination of the cerebellar specimen revealed a predominantly poorly differentiated tumor lacking glandular architecture, but with areas of squamous differentiation with focal p40 positivity (Fig. 2D). These features were also consistent with metastatic DEC.

### 3.2. Genomic findings

CGP was performed using the GenMineTOP® Cancer Genome Profiling System (Konica Minolta REALM, Inc., Tokyo, Japan), a hybrid-capture dual DNA-RNA assay that incorporates tumor-normal paired analysis. The DNA panel interrogates 737 cancer-associated genes, whereas the RNA panel detects fusions in 455 genes.^[10]^ Tumor purity was 50%, and the assay identified 46 genomic mutations, corresponding to a tumor mutational burden of 49.7 mutations per megabase (Table 1). A somatic *POLE* V411L mutation was detected, with a variant allele frequency of 63.9%, accompanied by pathogenic mutations in *PTEN*, *PIK3CA*, and *TP53* (Table 1).

## 4. Discussion

This case report describes a patient with advanced DEC harboring a *POLE* exonuclease domain mutation who presented with widespread metastases, including multiple brain lesions, and subsequently achieved durable disease control following multidisciplinary treatment including ICI-based therapy.

This study highlighted two significant aspects. First, this clinical scenario is exceptionally rare. Within DEC, *POLE*-mutated tumors account for approximately 12.4% of pooled cases,^[11]^ and most reports describe early stage disease; advanced-stage *POLE*-mutated DEC has rarely been documented.^[12]^ In contrast, MSI has been observed in approximately 44% of DEC cases.^[11]^ Prolonged survival has been reported with chemotherapy combined with ICI therapy in MMR-deficient DEC, which serves as a surrogate for MSI-high tumors,^[13]^ whereas comparable outcomes in *POLE*-mutated DEC have not been described. Brain metastases from EC are exceedingly uncommon, occurring in approximately 0.8% of the cases.^[14]^ Although a case of conventional EC with brain metastasis harboring a *POLE* mutation that responded to ICI therapy has been reported previously,^[15]^ no prior reports have described a case of DEC with both brain metastasis and a *POLE* mutation. Thus, the clinical behavior and therapeutic responsiveness of *POLE*-mutated DEC with central nervous system involvement remain undefined.

Second, the prognostic implications of this case report are noteworthy. Nearly half of patients with DEC present with advanced disease, and the 5-year overall survival for stage IV cases has been reported to be as low as 15%.^[4]^ In the present case, the biopsy specimen was predominantly undifferentiated endometrial carcinoma (UEC) with only a minor gland-forming component, a pattern typically associated with high clinical risk and consistent with data showing that even 20% UEC confers outcomes comparable to those of pure UEC.^[4]^

At the molecular level, the tumor harbored *TP53* mutations along with a pathogenic *POLE* exonuclease domain mutation; however, no alterations in the SWI/SNF complex were identified. According to TCGA, pathogenic *POLE* exonuclease domain mutations classify tumors into the favorable *POLE*-ultramutated group and are typically associated with low rates of recurrence and distant spread, even when *TP53* alterations co-occur.^[16]^ In contrast, our patient developed multiorgan metastases, including brain lesions, and demonstrated rapid early progression. Brain metastases in EC are associated with poor prognosis, with a median survival of approximately 6 months.^[14]^ Although the contribution of pembrolizumab cannot be distinguished from that of preceding chemotherapy and local therapies, the continued tumor regression observed during pembrolizumab maintenance therapy after completion of cytotoxic chemotherapy suggests that pembrolizumab may have contributed to the durable disease control, even in the setting of a UEC-predominant tumor with aggressive clinical features. The presence of a pathogenic *POLE* exonuclease domain mutation and high TMB may have contributed to the tumor’s sensitivity to immune checkpoint blockade and provides a plausible biological rationale for the sustained response.

This report has several limitations. As a single-case observation, these findings cannot be generalized to all patients with *POLE*-mutated DEC, and potential selection or publication biases should be acknowledged. In addition, because the patient received multimodal treatment, including surgery, radiotherapy, chemotherapy, and immunotherapy, the specific contribution of each modality to the clinical outcome could not be precisely determined; coexisting pathogenic *TP53*, *PTEN*, and *PIK3CA* alterations may also have influenced the clinical behavior and treatment response. Despite these limitations, the present case provides valuable insights into the potential role of comprehensive molecular profiling and ICI-containing multidisciplinary treatment in this tumor subtype.

To the best of our knowledge, this is one of the few reported cases of durable disease control following multidisciplinary treatment including ICI-based therapy in a patient with *POLE*-mutated DEC complicated by brain metastases. This case highlights the need for further research to clarify the potential contribution of immunotherapy and the clinical significance of *POLE* mutations in this rare and challenging disease.

## 5. Conclusion

*POLE*-mutated DEC with brain metastases is exceptionally rare, and evidence regarding the efficacy of ICI therapy in this context remains limited. This case demonstrates that even in the setting of aggressive disease, ICI therapy as part of a multidisciplinary treatment approach was associated with durable disease control, highlighting the potential therapeutic relevance of immunotherapy in this rare entity.

## Acknowledgments

The authors would like to thank Editage (www.editage.com) for providing English language editing assistance.

## Author contributions

**Conceptualization:** Takumi Ito, Jumpei Yoshida, Akari Iwakoshi, Keiji Sugiyama, Tomoko Inaba, Masashi Sanada, Chiyoe Kitagawa.

**Data curation:** Takumi Ito, Jumpei Yoshida, Akari Iwakoshi, Tomoko Inaba.

**Formal analysis:** Takumi Ito, Jumpei Yoshida, Keiji Sugiyama, Masashi Sanada, Chiyoe Kitagawa.

**Funding acquisition:** Takumi Ito, Jumpei Yoshida, Chiyoe Kitagawa.

**Investigation:** Takumi Ito, Jumpei Yoshida, Akari Iwakoshi, Keiji Sugiyama, Masashi Sanada.

**Methodology:** Takumi Ito, Jumpei Yoshida, Akari Iwakoshi, Keiji Sugiyama, Rieko Nishimura, Tomoko Inaba, Masashi Sanada, Chiyoe Kitagawa.

**Project administration:** Takumi Ito, Jumpei Yoshida, Chiyoe Kitagawa.

**Resources:** Takumi Ito, Jumpei Yoshida, Akari Iwakoshi, Keiji Sugiyama, Chiyoe Kitagawa.

**Software:** Takumi Ito, Keiji Sugiyama, Chiyoe Kitagawa.

**Validation:** Takumi Ito, Jumpei Yoshida, Keiji Sugiyama, Rieko Nishimura, Tomoko Inaba, Masashi Sanada, Chiyoe Kitagawa.

**Visualization:** Takumi Ito, Jumpei Yoshida, Akari Iwakoshi, Keiji Sugiyama, Rieko Nishimura, Masashi Sanada, Chiyoe Kitagawa.

**Writing – original draft:** Takumi Ito, Akari Iwakoshi.

**Writing – review & editing:** Takumi Ito, Jumpei Yoshida, Akari Iwakoshi, Keiji Sugiyama, Rieko Nishimura, Masashi Sanada, Chiyoe Kitagawa.

**Supervision:** Jumpei Yoshida, Akari Iwakoshi, Keiji Sugiyama, Rieko Nishimura, Tomoko Inaba, Masashi Sanada, Chiyoe Kitagawa.
